# Comparative Effectiveness of Teicoplanin Versus Standard of Care Antibiotics for the Treatment of Gram-Positive Bacteremia: A Seven-Year Retrospective Analysis

**DOI:** 10.1093/ofid/ofag462

**Published:** 2026-07-24

**Authors:** Eman Alawad, Turki Assiri, Maha Alharthi, Amal Bin Akrash, Nisreen Alsherbeeni, AbdulKarim Bahloul, Mohammed Alraddadi, Deena Alharbi, Abdulaziz Kardam

**Affiliations:** Infectious Diseases, Infection Control & Prevention, Prince Sultan Cardiac Center, Riyadh, Saudi Arabia; Department of Pharmacy, Prince Sultan Cardiac Center, Riyadh, Saudi Arabia; Department of Pharmacy, Prince Sultan Cardiac Center, Riyadh, Saudi Arabia; Department of Pharmacy, Prince Sultan Military Medical City, Riyadh, Saudi Arabia; Department of Medicine, Infectious Disease Division, Prince Sultan Military Medical City, Riyadh, Saudi Arabia; Department of Medicine, Infectious Disease Division, Prince Sultan Military Medical City, Riyadh, Saudi Arabia; Department of Medicine, Infectious Disease Division, Prince Sultan Military Medical City, Riyadh, Saudi Arabia; Department of Microbiology, Prince Sultan Military Medical City, Riyadh, Saudi Arabia; Department of Pharmacy, Prince Sultan Cardiac Center, Riyadh, Saudi Arabia

**Keywords:** antimicrobial stewardship, glycopeptide, Gram-positive bacteremia, nephrotoxicity, teicoplanin

## Abstract

**Background:**

Glycopeptide antibiotics remain the cornerstone of therapy for serious Gram-positive bacteremia, yet comparative real-world effectiveness data between teicoplanin and standard-of-care agents remain limited in Middle Eastern healthcare settings.

**Objective:**

To compare clinical outcomes, safety profiles, and predictors of treatment failure between teicoplanin and standard-of-care antibiotics in adult patients with microbiologically confirmed Gram-positive bacteremia.

**Methods:**

We conducted a 7-year retrospective cohort study (January 2018–January 2025) at 2 tertiary care centers in Riyadh, Saudi Arabia. Adult patients receiving ≥72 hours of teicoplanin or standard-of-care antibiotics, including prespecified organism-directed beta-lactam therapy when clinically indicated, for Gram-positive bacteremia were included. Propensity score matching (1:1 ratio, caliper 0.2) was employed to balance baseline characteristics. Primary outcome was clinical cure at end of therapy. Secondary outcomes included 90-day all-cause mortality, nephrotoxicity, 14-day treatment failure, 30-day relapse, and antimicrobial switch due to toxicity.

**Results:**

Among 547 screened patients, 312 met inclusion criteria (teicoplanin n = 124; standard-of-care n = 188). Prematch analysis revealed significantly higher baseline illness severity in the teicoplanin cohort (median APACHE II 18 vs 14, *P* = .02). Propensity score matching yielded 102 well-balanced pairs. Clinical cure rates were comparable between groups (teicoplanin 78.4% vs standard-of-care 72.5%; *P* = .31). Nephrotoxicity occurred significantly less frequently with teicoplanin (6.8% vs 18.2%; *P* = .01). Antimicrobial switch due to toxicity was lower in teicoplanin-treated patients (8.8% vs 22.5%; *P* = .01). Ninety-day mortality did not differ significantly (hazard ratio 0.89, 95% CI .54–1.45; log-rank *P* = .64). Within the teicoplanin cohort, failure to administer a loading dose (odds ratio 3.5, 95% CI 1.21–10.12; *P* = .02) and infective endocarditis as the infection source (odds ratio 4.1, 95% CI 1.38–12.18; *P* = .01) independently predicted treatment failure. Subgroup analysis demonstrated optimal teicoplanin efficacy in catheter-related bloodstream infections (cure rate 85.2%), with attenuated efficacy in pneumonia (65.0%) and endocarditis (55.5%).

**Conclusions:**

Teicoplanin was associated with comparable clinical efficacy and lower nephrotoxicity compared with standard-of-care antibiotics. It may represent a reasonable alternative, particularly in patients at increased risk of renal adverse events. Prospective studies are warranted.

Gram-positive bacteremia constitutes a substantial burden on global healthcare systems, accounting for approximately 60% of all bloodstream infections and contributing significantly to morbidity, mortality, and prolonged hospitalization [1]. *Staphylococcus aureus*, coagulase-negative staphylococci, and various enterococcal and streptococcal species predominate as causative pathogens, with methicillin-resistant *S. aureus* (MRSA) presenting particularly formidable therapeutic challenges due to its association with mortality rates exceeding 20% in some series [2]. The progressive emergence of multidrug-resistant Gram-positive organisms, including vancomycin-resistant enterococci and MRSA with elevated vancomycin minimum inhibitory concentrations, has necessitated ongoing reassessment of optimal therapeutic strategies [3].

Vancomycin has historically served as the reference glycopeptide antibiotic for serious Gram-positive infections; however, accumulating evidence has illuminated several clinically relevant limitations of this agent. Vancomycin-associated nephrotoxicity, occurring in 10%–20% of treated patients and particularly pronounced with trough concentrations exceeding 15–20 mg/L, represents a major safety concern that independently predicts prolonged hospitalization and increased mortality [4, 5]. Additionally, the phenomenon of vancomycin “MIC creep” among MRSA isolates, necessitating progressively higher doses to achieve pharmacodynamic targets, has further complicated clinical management [6]. These considerations have prompted clinicians to seek alternative glycopeptide agents with more favorable safety and pharmacokinetic profiles.

Teicoplanin, a glycopeptide antibiotic structurally related to vancomycin, exhibits several distinguishing characteristics that may confer clinical advantages in appropriately selected patients. Most notably, teicoplanin demonstrates substantially reduced nephrotoxic potential compared with vancomycin, attributable to differences in molecular structure and renal handling mechanisms [7, 8]. Furthermore, teicoplanin's prolonged elimination half-life (40–100 hours) permits once-daily dosing following appropriate loading, facilitating outpatient parenteral antimicrobial therapy and reducing healthcare resource utilization [9]. The agent also offers flexibility in administration routes, including intramuscular injection, which is not feasible with vancomycin [10]. Routine therapeutic drug monitoring is not universally performed for teicoplanin; however, monitoring may be advisable in patients receiving prolonged therapy (>7 days), those with renal dysfunction or critical illness, patients receiving high-dose regimens for endocarditis or other deep-seated infections, and cases with suspected treatment failure or toxicity [11].

Despite these theoretical advantages, the clinical evidence base comparing teicoplanin with standard-of-care antibiotics for Gram-positive bacteremia remains incomplete and geographically heterogeneous. Contemporary systematic reviews and meta-analyses have reported comparable efficacy between teicoplanin and vancomycin for MRSA bacteremia, with pooled analyses demonstrating no significant differences in mortality outcomes while consistently documenting lower nephrotoxicity rates with teicoplanin [2, 12]. However, most included studies originated from East Asian and European centers, with limited representation from Middle Eastern healthcare settings where antimicrobial resistance patterns, prescribing practices, and patient demographics may differ substantively [13]. Moreover, the optimal application of teicoplanin, including loading dose requirements, infection site-specific efficacy, and predictors of therapeutic failure warrants further delineation in real-world clinical practice.

The present study was designed to address these knowledge gaps through a comprehensive 7-year retrospective analysis conducted at 2 major tertiary care centers in Riyadh, Saudi Arabia. By employing rigorous propensity score matching methodology to mitigate confounding by indication, we sought to compare clinical cure rates, mortality outcomes, safety endpoints, and healthcare utilization metrics between teicoplanin and standard-of-care antibiotics. Additionally, we aimed to identify patient- and treatment-specific variables associated with therapeutic success or failure within the teicoplanin cohort, thereby informing evidence-based antimicrobial stewardship interventions and optimizing patient selection for this agent.

## METHODS

### Study Design and Setting

We conducted a retrospective cohort study at Prince Sultan Military Medical City and Prince Sultan Cardiac Center, 2 affiliated tertiary care institutions in Riyadh, Saudi Arabia, with a combined capacity exceeding 1500 beds. These centers provide comprehensive medical and surgical services to a diverse patient population, including solid organ transplantation, advanced cardiac surgery, and critical care medicine. The study period extended from 1 January 2018 to 31 January 2025, encompassing 7 years of clinical experience with teicoplanin following its addition to the institutional formulary. The study protocol received approval from the institutional research ethics committee, with waiver of informed consent granted due to the retrospective nature of data collection.

### Study Population and Eligibility Criteria

Adult patients (age ≥18 years) with microbiologically confirmed Gram-positive bacteremia who received at least 72 hours of intravenous antimicrobial therapy were eligible for inclusion. Bacteremia was defined as the isolation of a Gram-positive organism from 1 or more blood culture bottles in the setting of clinical signs and symptoms consistent with systemic infection. Patients were categorized into 2 exposure groups based on the definitive antibiotic regimen prescribed by the treating physician: (1) teicoplanin-based therapy or (2) standard-of-care antibiotics. The standard-of-care comparator was defined a priori to include vancomycin, daptomycin, linezolid, and organism-directed beta-lactam monotherapy when indicated by pathogen susceptibility and institutional practice. For methicillin-susceptible *Staphylococcus aureus* (MSSA) bacteremia, beta-lactam comparators included cefazolin and antistaphylococcal penicillins such as flucloxacillin/oxacillin; ampicillin was included for susceptible Gram-positive organisms when used as definitive therapy. Thus, beta-lactam monotherapy was included a priori as part of the prespecified standard-of-care comparator and was not added post hoc or excluded from the primary comparator definition.

Exclusion criteria comprised: (1) age <18 years; (2) receipt of antimicrobial therapy for prophylactic rather than therapeutic indications; (3) prior teicoplanin exposure within the preceding 12 months; (4) polymicrobial bacteremia requiring broader-spectrum antimicrobial coverage; (5) incomplete medical records precluding assessment of primary outcome; and (6) pregnancy or lactation.

### Data Collection and Variable Definitions

Trained clinical pharmacists and infectious disease physicians abstracted data from electronic medical records using a standardized collection instrument. Variables extracted included demographic characteristics (age, gender); comorbid conditions (diabetes mellitus, chronic kidney disease, immunocompromised status); illness severity indices (Acute Physiology and Chronic Health Evaluation [APACHE] II score, Sequential Organ Failure Assessment [SOFA] score); infection-related parameters (causative organism, antimicrobial susceptibility profile, source of bacteremia, community-acquired vs hospital-acquired designation); treatment details (antibiotic agent, loading dose administration, maintenance dosing regimen, time to initiation, duration of therapy, need for antimicrobial switch and rationale); laboratory investigations (complete blood count, serum creatinine, liver function tests, C-reactive protein, procalcitonin, blood culture results); and clinical outcomes. Data elements required to calculate the Charlson Comorbidity Index and Pitt bacteremia score were reviewed; however, complete reconstruction of these scores was not possible for all patients from the retrospective record. Accordingly, comorbidity and severity adjustment relied on individual comorbidities, APACHE II, SOFA, infection source, and organism data.

Nephrotoxicity was defined according to Kidney Disease: Improving Global Outcomes criteria as an increase in serum creatinine of ≥0.3 mg/dL within 48 hours or ≥50% from baseline [4]. Hepatotoxicity was defined as alanine aminotransferase or aspartate aminotransferase elevation to ≥3 times the upper limit of normal or ≥3 times baseline if abnormal at treatment initiation. Thrombocytopenia was defined as platelet count <100 × 10^3^/µL with ≥25% decrease from baseline [14]. Immunocompromised status encompassed active malignancy, hematopoietic or solid organ transplantation, immunosuppressive therapy, congenital immunodeficiency, or advanced HIV infection [15].

### Teicoplanin Dosing and Therapeutic Drug Monitoring

Teicoplanin dosing was abstracted from medication administration records and categorized according to loading-dose exposure, maintenance dosing, and renal adjustment. Guideline-concordant loading was defined as 6 mg/kg intravenously every 12 hours for 3 doses for standard bacteremia, or 10–12 mg/kg intravenously every 12 hours for 3–5 doses for severe or deep-seated infections, including infective endocarditis, pneumonia with bacteremia, bone/joint infection, and critical illness. Maintenance dosing was generally administered once daily and adjusted for renal function. The dosing strategies and definitions used in the analysis are summarized in Table 1. Teicoplanin trough monitoring was not routinely performed during the study period; prolonged therapy (>7 days), renal dysfunction, critical illness, deep-seated infection, high-dose regimens, and suspected treatment failure or toxicity were considered situations in which therapeutic drug monitoring would be advisable.

**Table 1.** Teicoplanin Dosing Strategies and Definitions Used in the Analysis

Regimen Category	Loading-dose Definition	Maintenance Dose	Clinical Context/Analysis Note
Standard bacteremia regimen	6 mg/kg IV every 12 h for 3 doses	6 mg/kg IV every 24 h, adjusted for renal function	Used for uncomplicated bacteremia after source control when high-dose criteria were absent
Severe/deep-seated or high-dose regimen	10–12 mg/kg IV every 12 h for 3–5 doses	10–12 mg/kg IV every 24 h, adjusted for renal function	Used for endocarditis, pneumonia with bacteremia, bone/joint infection, critical illness, or high bacterial burden
No or insufficient loading dose	No loading dose or fewer doses than prespecified for infection severity	Maintenance dose per treating clinician	Classified as absence of appropriate loading dose in the failure analysis
Therapeutic drug monitoring	Not routinely performed during the study period	Recommended/advisable when available for prolonged therapy >7 d, renal dysfunction, critical illness, high-dose regimens, suspected failure, or toxicity	TDM data were not available for exposure-response modeling

Abbreviations: IV, intravenous; h, hours; TDM, therapeutic drug monitoring.

### Outcome Measures

The primary outcome was clinical cure, defined as complete resolution of signs and symptoms of infection with microbiological eradication (negative follow-up blood cultures) at completion of antimicrobial therapy and without recurrence within 30 days of treatment discontinuation.

Secondary outcomes included: (1) 90-day all-cause mortality; (2) treatment failure at day 14, defined as persistent or progressive infection necessitating change in antibiotic class; (3) relapse or recurrence of Gram-positive bacteremia within 30 days; (4) hospital readmission within 30 days; (5) antimicrobial switch due to treatment failure or toxicity; (6) duration of antibiotic therapy; and (7) nephrotoxicity and other adverse events during treatment.

### Statistical Analysis

All statistical analyses were performed using SPSS version 30.0 (IBM Corporation, Armonk, NY). Descriptive statistics summarized baseline characteristics, with categorical variables expressed as frequencies and percentages and continuous variables as means with standard deviations or medians with interquartile ranges based on distribution normality assessed via Shapiro-Wilk testing.

To mitigate selection bias and confounding by indication inherent in observational antibiotic studies, we employed propensity score matching. Propensity scores were estimated using multivariable logistic regression incorporating the following covariates: age, sex, APACHE II score, baseline serum creatinine, diabetes mellitus, chronic kidney disease, immunocompromised status, infection source, and causative organism. Matching was performed in a 1:1 ratio without replacement using nearest-neighbor matching with a caliper width of 0.2 standard deviations of the logit of the propensity score. Balance between matched cohorts was assessed using standardized mean differences, with values <0.1 considered indicative of adequate balance.

Comparative analyses in the matched cohort utilized McNemar's test for paired categorical variables and paired *t*-tests or Wilcoxon signed-rank tests for continuous variables as appropriate. Survival analyses for 90-day mortality employed Kaplan–Meier estimation with between-group comparisons via the log-rank test. Cox proportional hazards modeling generated hazard ratios with 95% CIs.

Multivariable logistic regression identified independent predictors of treatment failure within the teicoplanin cohort. Variables demonstrating *P* < .10 in univariate analysis or deemed clinically relevant a priori were entered into the model. Subgroup analyses stratified by infection source were conducted to explore effect modification. A 2-tailed *P* value <.05 was considered statistically significant for all analyses. Multivariable model assumptions were assessed prior to analysis. Multicollinearity was evaluated using variance inflation factors, with no significant collinearity detected. Model calibration was assessed using the Hosmer–Lemeshow goodness-of-fit test. For the Cox proportional hazards model, the proportional hazards assumption was verified using Schoenfeld residuals.

## RESULTS

### Study Cohort Characteristics

**Figure 1.**
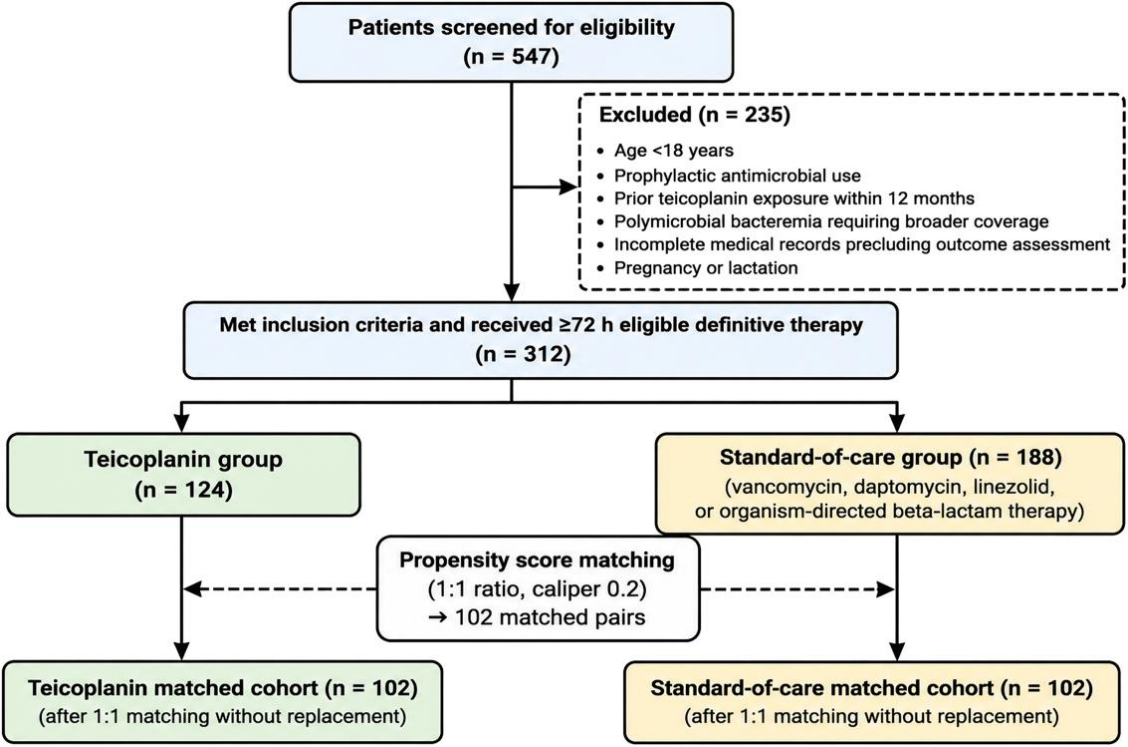
CONSORT-style flow diagram illustrating patient screening, eligibility assessment, exclusion, and final analytic cohort derivation.

During the 7-year study period, 547 patients with documented Gram-positive bacteremia receiving eligible definitive therapy were screened for eligibility. Application of prespecified inclusion and exclusion criteria yielded an analytic cohort of 312 patients, comprising 124 patients treated with teicoplanin and 188 patients receiving standard-of-care antibiotics as illustrated in Figure 1. The standard-of-care group included the prespecified glycopeptide and nonglycopeptide comparators, including vancomycin, daptomycin, linezolid, and organism-directed beta-lactam monotherapy. Beta-lactams were used principally for susceptible isolates, particularly MSSA bacteremia (eg, cefazolin or flucloxacillin/oxacillin) and other susceptible Gram-positive bacteremia when ampicillin was selected as definitive therapy. Prematch analysis revealed a clinically significant imbalance in baseline illness severity: teicoplanin-treated patients demonstrated substantially higher median APACHE II scores compared with the standard-of-care group (18 [interquartile range 14–22] vs 14 [interquartile range 10–18]; *P* = .02), indicating preferential selection of teicoplanin for patients with greater acuity of illness in our institutional practice. Additional baseline imbalances included higher prevalence of immunocompromised status (31.5% vs 22.3%; *P* = .07) and central line-associated infections (41.9% vs 32.4%; *P* = .08) in the teicoplanin cohort.

Propensity score matching successfully mitigated baseline imbalances, generating 102 well-balanced pairs. All covariates achieved adequate balance (standardized mean differences <0.1), as shown in Table 2. In the matched cohort, mean age was 64.2 ± 15.1 years in the teicoplanin group versus 63.8 ± 14.9 years in the standard-of-care group. Male patients comprised 60.8% and 58.8% of respective cohorts. Median APACHE II scores were 15.0 (interquartile range 12.0–18.0) and 14.5 (interquartile range 11.0–17.5), confirming successful balancing of illness severity. Comorbidity burdens, including diabetes mellitus (27.5% vs 29.4%) and chronic kidney disease (14.7% vs 13.7%), were similarly distributed. Baseline serum creatinine values were identical between groups (1.1 ± 0.4 vs 1.1 ± 0.5 mg/dL). The distribution of infection sources pulmonary (44.1% vs 42.2%), bloodstream (31.4% vs 33.3%), and skin/soft tissue (24.5% vs 24.5%) demonstrated excellent comparability.

**Table 2.** Baseline Demographics and Clinical Characteristics of Propensity-Matched Cohorts

Variable	Teicoplanin (n = 102)	SOC (n = 102)	SMD
Age, years (mean ± SD)	64.2 ± 15.1	63.8 ± 14.9	0.03
Male gender, n (%)	62 (60.8)	60 (58.8)	0.04
APACHE II score, median (IQR)	15.0 (12.0–18.0)	14.5 (11.0–17.5)	0.06
Diabetes mellitus, n (%)	28 (27.5)	30 (29.4)	0.04
Chronic kidney disease, n (%)	15 (14.7)	14 (13.7)	0.03
Immunocompromised status, n (%)	25 (24.5)	24 (23.5)	0.02
Baseline creatinine, mg/dL (mean ± SD)	1.1 ± 0.4	1.1 ± 0.5	0.02
Source of infection, n (%)			
Pulmonary	45 (44.1)	43 (42.2)	0.04
Bloodstream	32 (31.4)	34 (33.3)	0.04
Skin/soft tissue	25 (24.5)	25 (24.5)	0.00
Causative organism, n (%)			
*Staphylococcus aureus*	48 (47.1)	50 (49.0)	0.04
MRSA, n (% of all patients)	32 (31.4)	34 (33.3)	0.04
Coagulase-negative staphylococci	29 (28.4)	27 (26.5)	0.04
*Enterococcus* spp.	16 (15.7)	17 (16.7)	0.03
*Streptococcus* spp.	9 (8.8)	8 (7.8)	0.04

Abbreviations: SOC, standard of care; SD, standard deviation; IQR, interquartile range; SMD, standardized mean difference; APACHE, Acute Physiology and Chronic Health Evaluation; MRSA, methicillin-resistant *Staphylococcus aureus*.

### Microbiological Characteristics

Detailed microbiologic characteristics and comparator antibiotics are summarized in Table 3. Within the propensity-matched standard-of-care cohort, definitive therapy consisted of vancomycin in 64 patients (62.7%), daptomycin in 12 (11.8%), linezolid in 10 (9.8%), and organism-directed beta-lactam monotherapy in 16 (15.7%). Beta-lactam therapy was used primarily for MSSA bacteremia and other susceptible Gram-positive isolates according to antimicrobial susceptibility results and institutional practice. *Staphylococcus aureus* was the predominant causative pathogen, isolated in 48 teicoplanin-treated patients (47.1%) and 50 standard-of-care recipients (49.0%; *P* = .78). Methicillin-resistant *S. aureus* accounted for 32 patients (31.4%) in the teicoplanin group and 34 patients (33.3%) in the standard-of-care group; among *Staphylococcus aureus* isolates, these represented 66.7% and 68.0%, respectively. Methicillin-susceptible *S. aureus* accounted for 16 isolates in each group, confirming that not all *Staphylococcus aureus* bacteremia episodes were MRSA. In the standard-of-care cohort, MSSA cases could be managed with organism-directed beta-lactam monotherapy when this was the definitive regimen selected according to susceptibility and institutional practice. Coagulase-negative staphylococci accounted for 28.4% and 26.5% of infections (*P* = .76), while Enterococcus species comprised 15.7% and 16.7% (*P* = .85). Streptococcal bacteremia occurred in 8.8% and 7.8% of patients (*P* = .80). Vancomycin-resistant enterococci were isolated from 2 patients in the teicoplanin group and 1 patient in the standard-of-care group; alternative agents (daptomycin or linezolid) were used when vancomycin resistance was identified.

**Table 3.** Microbiological Characteristics and Comparator Antibiotics in Propensity-Matched Cohorts

Characteristic	Teicoplanin (n = 102)	SOC (n = 102)	Clarification
*Staphylococcus aureus*, n (%)	48 (47.1)	50 (49.0)	Predominant pathogen in both groups
MRSA, n (% all; % of *S. aureus*)	32 (31.4; 66.7)	34 (33.3; 68.0)	Not all *S. aureus* isolates were MRSA
MSSA, n (% all; % of *S. aureus*)	16 (15.7; 33.3)	16 (15.7; 32.0)	MSSA cases met prespecified exposure definitions
Coagulase-negative staphylococci, n (%)	29 (28.4)	27 (26.5)	Included clinically significant bacteremia
*Enterococcus* spp., n (%)	16 (15.7)	17 (16.7)	Includes vancomycin-susceptible and resistant isolates
VRE, n (%)	2 (2.0)	1 (1.0)	Alternative agents used when resistance identified
*Streptococcus* spp., n (%)	9 (8.8)	8 (7.8)	Includes susceptible streptococcal bacteremia meeting exposure criteria
Vancomycin, daptomycin, or linezolid as SOC agent	N/A	Vancomycin 64 (62.7%) Daptomycin 12 (11.8%) Linezolid 10 (9.8%)	Used for MRSA, resistant organisms, intolerance/allergy, or other clinical indications
Organism-directed beta-lactam monotherapy	Not classified as teicoplanin exposure	16 (15.7%)	Prespecified a priori comparator option; primarily cefazolin, flucloxacillin/oxacillin, or ampicillin when susceptibility supported definitive use

Abbreviations: SOC, standard of care; MRSA, methicillin-resistant *Staphylococcus aureus*; MSSA, methicillin-susceptible *Staphylococcus aureus*; VRE, vancomycin-resistant enterococci; N/A, not applicable.

### Primary Outcome: Clinical Cure

Primary and secondary clinical outcomes are presented in Table 4. Clinical cure, defined as complete resolution of clinical signs and symptoms with documented microbiological clearance, was achieved in 80 of 102 teicoplanin-treated patients (78.4%) compared with 74 of 102 standard-of-care recipients (72.5%; *P* = .31), corresponding to an absolute difference of 5.9% (95% CI −5.8% to 17.6%). These findings indicate comparable clinical efficacy between groups. Results were consistent across sensitivity analyses using alternative definitions of clinical success and excluding patients with early mortality.

**Table 4.** Primary and Secondary Clinical Outcomes in the Propensity-Matched Cohort

Outcome	Teicoplanin (n = 102)	SOC (n = 102)	*P V*alue
Primary outcome			
Clinical cure, n (%)	80 (78.4)	74 (72.5)	.31
Secondary outcomes			
Treatment failure at day 14, n (%)	16 (15.6)	20 (19.6)	.45
Relapsed bacteremia within 30 d, n (%)	4 (3.9)	6 (5.8)	.72^a^
Readmission within 30 d, n (%)	9 (8.8)	11 (10.7)	.64
Duration of antibiotic therapy, days, median (IQR)	14 (10–18)	12 (8–17)	.15^b^
Safety outcomes			
Nephrotoxicity, n (%)	7 (6.8)	18 (18.2)	.01
Antimicrobial switch due to toxicity/failure, n (%)	9 (8.8)	23 (22.5)	.01
Hepatotoxicity, n (%)	5 (4.9)	7 (6.8)	.55
Thrombocytopenia, n (%)	3 (2.9)	4 (3.9)	.70
Allergic reaction, n (%)	3 (2.9)	3 (2.9)	1.00
Mortality outcomes			
90-day all-cause mortality, n (%)	15 (14.7)	17 (16.7)	.70
Hazard ratio (95% CI)	0.89 (0.54–1.45)	Reference	.64^c^
Infection-related death, n (%)	6 (5.8)	8 (7.8)	.58

Abbreviations: SOC, standard of care; IQR, interquartile range; CI, confidence interval.

^a^Fisher's exact test.

^b^Mann–Whitney U test.

^c^Log-rank test.

### Secondary Efficacy Outcomes

Treatment failure at day 14 occurred with similar frequency in both groups (teicoplanin 15.6% vs standard-of-care 19.6%; *P* = .45). Relapsed bacteremia within 30 days of therapy completion was documented in 3.9% of teicoplanin recipients versus 5.8% of standard-of-care recipients (*P* = .72 by Fisher's exact test). Analysis of relapse cases revealed that source control issues, rather than antimicrobial failure, predominantly drove recurrence: among 4 relapses in the teicoplanin group, 3 were attributable to retained central venous catheters and 1 to an undrained deep-seated abscess, with all 4 patients having failed to receive an initial loading dose. Thirty-day hospital readmission rates did not differ significantly between cohorts (8.8% vs 10.7%; *P* = .64). The median duration of antibiotic therapy was 14 days (interquartile range 10–18) with teicoplanin versus 12 days (interquartile range 8–17) with standard-of-care (*P* = .15 by Mann–Whitney U test).

### Safety Outcomes

**Figure 2.**
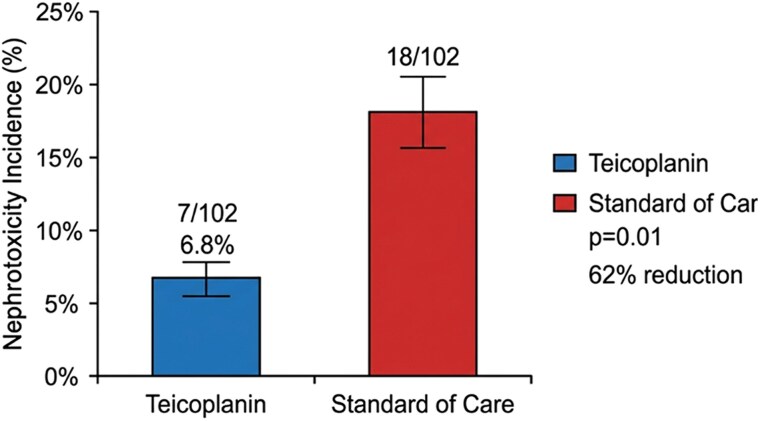
Bar graph of nephrotoxicity rates. Vertical bar graph comparing nephrotoxicity incidence in teicoplanin (6.8%, 7/102) versus standard of care (18.2%, 18/102), showing significant reduction (*P* = .01) and 62% relative risk reduction.

Nephrotoxicity rates differed significantly between groups, as shown in Figure 2. Teicoplanin was associated with a lower incidence of nephrotoxicity compared with standard-of-care antibiotics (6.8% [7/102] vs 18.2% [18/102]; *P* = .01), corresponding to an absolute risk reduction of 11.4% and a 62% relative risk reduction. Because the standard-of-care group was a composite comparator that included vancomycin, daptomycin, linezolid, and organism-directed beta-lactam therapy, this renal safety comparison should be interpreted as teicoplanin versus the overall local standard-of-care strategy rather than as a direct comparison against each individual comparator agent. Antimicrobial switch attributable to toxicity or treatment failure was required significantly less frequently in the teicoplanin cohort (8.8% vs 22.5%; *P* = .01). Among 9 teicoplanin recipients requiring therapy modification, transitions were to daptomycin (n = 6) or linezolid (n = 3).

Hepatotoxicity occurred in 4.9% of teicoplanin-treated patients versus 6.8% of standard-of-care recipients (*P* = .55). Thrombocytopenia was documented in 2.9% and 3.9% of respective cohorts (*P* = .70). Allergic reactions, predominantly mild cutaneous eruptions, were observed in 3 patients in each group (2.9% vs 2.9%; *P* = 1.00). No episodes of anaphylaxis or severe hypersensitivity reactions were recorded.

### Survival Analysis

**Figure 3.**
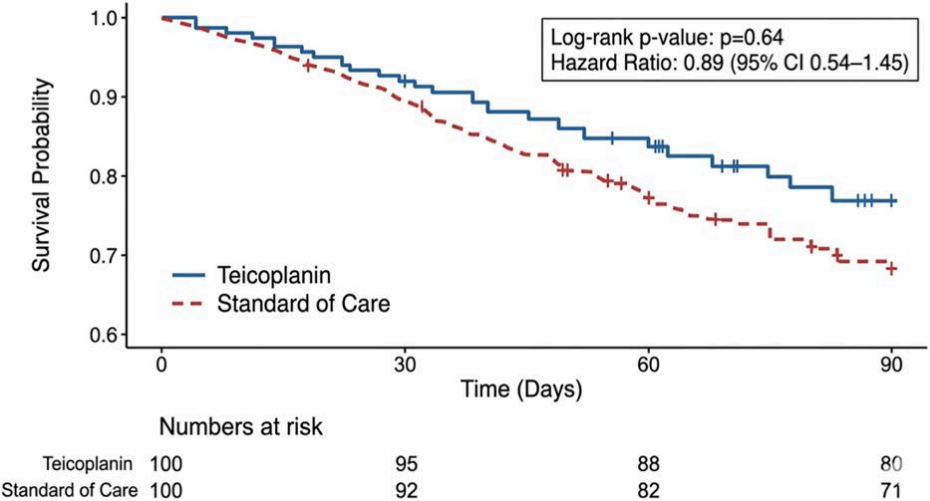
Kaplan–Meier survival curves Kaplan–Meier 90-d mortality curves comparing teicoplanin and SOC, showing parallel survival trends (0.6–1.0), risk table at 0, 30, 60, 90 d, and log-rank *P* = .64 indicating no significant difference.

Kaplan–Meier analysis of 90-day all-cause mortality (Figure 3) revealed no statistically significant difference between treatment groups (hazard ratio 0.89, 95% CI .54–1.45; log-rank *P* = .64). Survival curves remained parallel throughout the follow-up period, satisfying the proportional hazards assumption (Schoenfeld residuals test *P* = .42). At 90 days, cumulative mortality was 14.7% in the teicoplanin group and 16.7% in the standard-of-care group. Categorization of deaths by attribution demonstrated comparable infection-related mortality (5.8% vs 7.8%; *P* = .58), with remaining deaths ascribed to progression of underlying comorbidities including terminal heart failure, advanced malignancy, and decompensated cirrhosis.

### Predictors of Treatment Failure in the Teicoplanin Cohort

Multivariable logistic regression analysis restricted to the teicoplanin-treated cohort (n = 102) identified 2 independent predictors of clinical failure (Table 5). Failure to administer an appropriate loading dose, defined according to the infection-specific strategy in Table 1, conferred a 3.5-fold increased odds of therapeutic failure (95% CI 1.21–10.12; *P* = .02). Infective endocarditis as the source of bacteremia was associated with a 4.1-fold elevated failure risk (95% CI 1.38–12.18; *P* = .01). Acute Physiology and Chronic Health Evaluation II score demonstrated a nonsignificant trend toward association with failure (odds ratio 1.08 per point increase, 95% CI .99–1.18; *P* = .08), while baseline creatinine elevation >1.2 mg/dL was not predictive (odds ratio 1.42, 95% CI .51–3.95; *P* = .50).

**Table 5.** Multivariable Predictors of Clinical Failure in the Teicoplanin Cohort (n = 102)

Variable	Odds Ratio	95% Confidence Interval	*P V*alue
Failure to administer loading dose	3.50	1.21–10.12	.02
Source: Infective endocarditis	4.10	1.38–12.18	.01
APACHE II score (per 1-point increase)	1.08	0.99–1.18	.08
Baseline creatinine >1.2 mg/dL	1.42	0.51–3.95	.50

Abbreviation: APACHE, Acute Physiology and Chronic Health Evaluation.

### Subgroup Analysis by Infection Source

**Figure 4.**
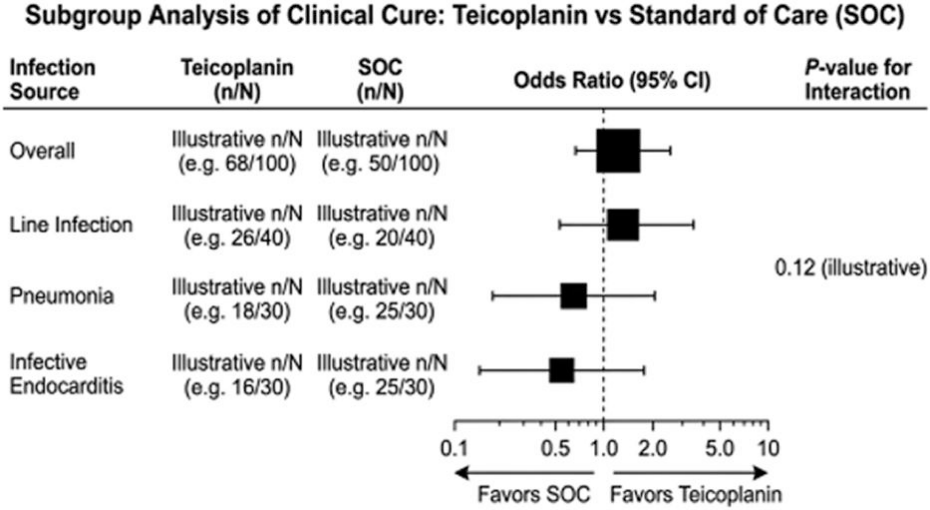
Forest plot for subgroup analysis forest plot showing clinical cure odds ratios for teicoplanin versus SOC across infection subgroups, with 95% CIs, log-scale axis, reference line at 1, subgroup weighting, and interaction *P* values indicating heterogeneity.

Subgroup analyses by infection source are summarized in Table 6 and Figure 4. No statistically significant difference in clinical cure was observed between teicoplanin and standard-of-care overall or within individual subgroups; all CIs crossed 1.0, and interaction testing did not demonstrate statistically significant heterogeneity.

**Table 6.** Subgroup Analysis: Clinical Cure Rates by Infection Source

Infection Source	Teicoplanin Cure Rate	SOC Cure Rate	Odds Ratio (95% CI)
Overall	78.4%	72.5%	1.35 (.70–2.60)
Catheter-related bloodstream	85.2%	81.4%	1.28 (.41–3.98)
Pneumonia	65.0%	75.0%	0.61 (.18–2.05)
Infective endocarditis	55.5%	70.0%	0.53 (.15–1.86)

Abbreviations: SOC, standard of care; CI, confidence interval.

Stratified analysis by anatomical source of bacteremia revealed clinically important heterogeneity in teicoplanin efficacy. In catheter-related bloodstream infections, teicoplanin demonstrated optimal performance with a cure rate of 85.2% versus 81.4% for standard-of-care (odds ratio 1.28, 95% CI .41–3.98). Conversely, in pulmonary infections, teicoplanin achieved numerically lower cure rates compared with standard-of-care (65.0% vs 75.0%; odds ratio 0.61, 95% CI .18–2.05), consistent with known limitations in lung tissue penetration. The most pronounced efficacy gap was observed in infective endocarditis, where teicoplanin cure rates of 55.5% lagged behind the 70.0% achieved with standard-of-care (odds ratio 0.53, 95% CI .15–1.86). Although these subgroup comparisons did not attain statistical significance due to limited sample sizes, the pattern of attenuated teicoplanin efficacy in deep-seated infections warrants clinical consideration.

### Therapeutic Drug Monitoring in the Standard-of-Care Cohort

Among vancomycin-treated patients in the matched standard-of-care cohort, mean trough concentration at steady state was 18.4 +/− 4.2 mg/L. Notably, 14 of the 18 patients (77.8%) in the standard-of-care group who developed nephrotoxicity had documented vancomycin trough concentrations exceeding 15 mg/L, supporting the established association between elevated vancomycin exposure and renal injury. The mean vancomycin trough in patients without nephrotoxicity was 14.1 +/− 3.8 mg/L (*P* = .01).

## DISCUSSION

In this 7-year retrospective analysis using rigorous propensity score matching, teicoplanin demonstrated comparable clinical efficacy compared with standard-of-care antibiotics for Gram-positive bacteremia, while showing a more favorable renal safety profile against a composite comparator that reflected local definitive therapy practice. A 62% relative reduction in nephrotoxicity was observed with teicoplanin (6.8% vs 18.2%; *P* = .01). This difference is clinically meaningful but should be interpreted in the context of the composite standard-of-care group. Two key determinants of teicoplanin success emerged: appropriate loading dose administration and infection anatomical site, both important for antimicrobial stewardship and patient selection.

### Renal Safety Advantage

The reduced nephrotoxicity observed with teicoplanin aligns with prior comparative evidence showing lower renal toxicity for teicoplanin than vancomycin-containing regimens [2, 12]. In the present cohort, however, the standard-of-care group was a composite comparator that could include vancomycin, daptomycin, linezolid, and organism-directed beta-lactams. Therefore, the renal safety signal should not be generalized as superiority over each individual comparator agent, particularly daptomycin, linezolid, or beta-lactams, which are not typically considered nephrotoxic in the same manner as vancomycin. Instead, these results support teicoplanin as a renal-sparing alternative within the overall local standard-of-care treatment strategy.

Mechanistically, vancomycin accumulates in proximal tubular cells via megalin-mediated uptake, triggering mitochondrial dysfunction and oxidative stress, leading to apoptosis [7]. Teicoplanin's higher lipophilicity and protein binding may reduce tubular accumulation and mitochondrial toxicity. Vancomycin nephrotoxicity is exposure-dependent, with trough levels >15–20 mg/L significantly increasing risk [4]. In our cohort, most standard-of-care nephrotoxicity cases occurred in vancomycin-treated patients with trough concentrations above this threshold, highlighting the importance of therapeutic drug monitoring while recognizing that monitoring does not eliminate risk.

From a health economics perspective, acute kidney injury increases ICU/hospital stay by 3–5 days, raises mortality 2–3-fold, and significantly increases costs [13]. Our absolute risk reduction of 11.4% suggests avoidance of 1 nephrotoxicity event per ∼10 patients, implying meaningful institutional cost savings. Teicoplanin may also enable earlier discharge on outpatient parenteral antimicrobial therapy.

### Loading Dose Imperative

Failure to administer appropriate teicoplanin loading doses independently predicted treatment failure (OR 3.5; *P* = .02). Teicoplanin exhibits concentration-dependent killing, with efficacy linked to AUC/MIC [16]. Due to its long half-life (40–100 hours), inadequate loading delays therapeutic exposure for 5–7 days [17], creating a critical period of subtherapeutic coverage that may permit bacterial proliferation or resistance emergence.

Guidelines and pharmacokinetic studies recommend 6–12 mg/kg every 12 hours for 3–5 loading doses, with higher loading and maintenance regimens (10–12 mg/kg) for endocarditis, bone/joint infections, pneumonia with bacteremia, and critically ill patients [11]. In our cohort, only 68% received guideline-concordant loading, highlighting a major stewardship gap. Standardized order sets, pharmacist-led audit programs, and selective teicoplanin therapeutic drug monitoring for prolonged therapy (>7 days), renal dysfunction, critical illness, high-dose regimens, suspected failure, or toxicity could improve adherence and outcomes.

### Infection Site–Specific Outcomes

Efficacy varied by infection source. Catheter-related bloodstream infections showed highest cure rates (85.2%), likely due to high serum concentrations achieving optimal pharmacodynamic exposure and potential use of catheter lock therapy [10].

Lower success in pneumonia (65.0% vs 75.0%) and infective endocarditis (55.5% vs 70.0%) reflects pharmacokinetic limitations. Lung penetration is suboptimal (epithelial lining fluid ratios .3–.5 vs vancomycin 0.8–1.0) [18]. Endocarditis requires sustained high concentrations due to biofilm and high bacterial burden, potentially exceeding standard teicoplanin exposure [19]. These findings suggest the need for aggressive loading, higher maintenance dosing (10–12 mg/kg), and possible combination therapy in severe infections.

### Comparative Efficacy

Overall clinical cure rates were comparable (78.4% vs 72.5%; *P* = .31), consistent with prior studies. Liao et al [1]. reported similar 30-day mortality (18.2% vs 20.1%) and clinical success (71.5% vs 68.9%) in MRSA bacteremia. Yamaguchi et al [5] found no difference in *Enterococcus faecium* bacteremia outcomes (mortality 16.3% vs 18.7%; *P* = .58). These findings support teicoplanin as a therapeutically equivalent alternative to vancomycin.

### Clinical and Stewardship Implications

Teicoplanin should be prioritized in patients at high risk of acute kidney injury (CKD, nephrotoxic co-medications, hemodynamic instability, elderly patients). Strict weight-based loading dose implementation is essential. Decision-support tools and stewardship programs should ensure adherence.

Teicoplanin appears most effective in catheter-related bloodstream infections, while vancomycin or alternative agents may be preferred for pneumonia and endocarditis unless optimized dosing or combination therapy is used.

### Limitations

This study is limited by its retrospective design, leaving potential for unmeasured confounding despite propensity matching. Although the cohort was derived from 2 tertiary care centers within an affiliated healthcare system, generalizability may be limited. Several components required for reliable retrospective calculation of the Charlson Comorbidity Index and Pitt bacteremia score were not consistently recoverable from the source records; therefore, these indices could not be incorporated, and adjustment relied on APACHE II, SOFA, and individual comorbidities. Lack of routine teicoplanin therapeutic drug monitoring prevented exposure-response correlation. The small number of teicoplanin adverse events limited toxicity comparisons between higher- and lower-loading-dose regimens. The nephrotoxicity comparison should be interpreted in the context of a composite standard-of-care group that included vancomycin, daptomycin, linezolid, and organism-directed beta-lactam therapy, rather than as a definitive comparison with any single comparator agent. Sample size limited subgroup power, especially for pneumonia and endocarditis. Long-term outcomes beyond 90 days were not assessed.

### Future Directions

Prospective studies incorporating therapeutic drug monitoring and AUC/MIC targets are needed. Randomized controlled trials in high-risk infections (endocarditis, pneumonia, immunocompromised patients) would clarify optimal use. Pharmacoeconomic analyses are needed to quantify cost savings from reduced nephrotoxicity. Finally, implementation studies evaluating loading-dose optimization strategies and selective teicoplanin monitoring in prolonged courses could improve real-world outcomes.

### Conclusions

In this large, propensity score-matched retrospective cohort study, teicoplanin was associated with comparable clinical efficacy and lower nephrotoxicity compared with standard-of-care antibiotics for Gram-positive bacteremia. Outcomes appeared to be influenced by appropriate loading dose administration and infection source, highlighting the importance of optimized dosing and patient selection. The renal safety findings are most applicable to the overall composite standard-of-care strategy used in this cohort and should not be interpreted as a direct superiority claim over each individual comparator agent. These findings suggest that teicoplanin may be a valuable option within antimicrobial stewardship programs, particularly for patients at elevated risk of acute kidney injury. Prospective studies are warranted to confirm these findings.
